# Exploring causal mechanisms of psychosis risk

**DOI:** 10.1016/j.neubiorev.2024.105699

**Published:** 2024-07

**Authors:** Dominic Oliver, Edward Chesney, Alexis E. Cullen, Cathy Davies, Amir Englund, George Gifford, Sarah Kerins, Paris Alexandros Lalousis, Yanakan Logeswaran, Kate Merritt, Uzma Zahid, Nicolas A. Crossley, Robert A. McCutcheon, Philip McGuire, Paolo Fusar-Poli

**Affiliations:** a Department of Psychiatry, University of Oxford, Oxford, UK; b NIHR Oxford Health Biomedical Research Centre, Oxford, UK; c OPEN Early Detection Service, Oxford Health NHS Foundation Trust, Oxford, UK; d Early Psychosis: Interventions and Clinical-Detection (EPIC) Lab, Department of Psychosis Studies, Institute of Psychiatry, Psychology & Neuroscience, King's College London, London, UK; e Department of Psychosis Studies, Institute of Psychiatry, Psychology & Neuroscience, King’s College London, London, UK; f Addictions Department, Institute of Psychiatry, Psychology and Neuroscience, King’s College London, 4 Windsor Walk, London SE5 8AF, UK; g Department of Clinical Neuroscience, Karolinska Institutet, Sweden; h Department of Neuroimaging, Institute of Psychiatry, Psychology & Neuroscience, King's College London, London, UK; i Department of Psychiatry and Psychotherapy, Ludwig-Maximilian-University Munich, Munich, Germany; j Department of Biostatistics & Health Informatics, King’s College London, London, UK; k Division of Psychiatry, Institute of Mental Health, UCL, London, UK; l Department of Psychology, King’s College London, London, UK; m Department of Psychiatry, School of Medicine, Pontificia Universidad Católica de Chile, Chile; n Oxford Health NHS Foundation Trust, Oxford, UK; o Department of Brain and Behavioral Sciences, University of Pavia, Pavia, Italy; p OASIS Service, South London and Maudsley NHS Foundation Trust, London SE11 5DL, UK

**Keywords:** Psychosis, Prevention, Risk factors, Neurobiology, Neurodevelopment, Dopamine, Glutamate

## Abstract

Robust epidemiological evidence of risk and protective factors for psychosis is essential to inform preventive interventions. Previous evidence syntheses have classified these risk and protective factors according to their strength of association with psychosis. In this critical review we appraise the distinct and overlapping mechanisms of 25 key environmental risk factors for psychosis, and link these to mechanistic pathways that may contribute to neurochemical alterations hypothesised to underlie psychotic symptoms. We then discuss the implications of our findings for future research, specifically considering interactions between factors, exploring universal and subgroup-specific factors, improving understanding of temporality and risk dynamics, standardising operationalisation and measurement of risk and protective factors, and developing preventive interventions targeting risk and protective factors.

## Highlights

• Many risk factors for psychosis have shared causal mechanisms.
• Many factors are associated with psychosocial stress.
• Other risk factors may more directly impact core neurobiological mechanisms.
• Combined measurement of these factors may improve prevention efforts.
• More research is needed to understand the complexities of risk/protective factors.

## 1 Introduction

Individuals with psychosis have high personal burden (Estradé et al., 2023, Fusar-Poli et al., 2022), poorer functioning (Gebreegziabhere et al., 2022) and shorter life expectancy (Charlson et al., 2018, Chesney et al., 2014) than those in the general population. Reducing the number of people who experience difficulties due to the disorder through primary preventative interventions is therefore an important goal (Fusar-Poli et al., 2021, Shah et al., 2022, Uhlhaas et al., 2023). These approaches can be implemented through universal (interventions targeting the general population), selective (interventions targeting individuals or groups that have increased risk due to certain characteristics) and indicated (interventions targeting individuals with increased risk due to detectable signs and symptoms) prevention approaches (Fusar-Poli et al., 2021). Worldwide, indicated approaches are predominantly implemented through the clinical high risk for psychosis (CHR-P) construct (Fusar-Poli et al., 2020, Fusar-Poli et al., 2021, Kotlicka-Antczak et al., 2020). 22% of individuals meeting CHR-P criteria will develop a psychotic disorder within two years of presenting to services (Fusar-Poli et al., 2020), compared to 0.43% in the general population (Fusar-Poli et al., 2016). As such, it is the psychosis risk factor with the greatest strength of evidence, with 10.9–12.3% of all new psychosis cases prevented if these approaches were 100% effective (Dragioti et al., 2022, Oliver et al., 2022).

The accumulation of risk factors throughout an individual’s life may partly explain the onset of the disorder (Oliver et al., 2019a). A better understanding of risk factors would enhance the detection of individuals at risk, facilitate accurate prognosis of clinical outcomes and target effective interventions. Detecting individuals at risk may be informed by assessing exposure to risk and protective factors for psychosis (Oliver et al., 2020b). Greater understanding of the mechanisms of risk and protective factors for psychosis would inform prognostication by providing key measures for clinical prediction models, particularly in combination with other data sources to improve model performance (Coutts et al., 2023). Targeted preventive interventions can similarly be developed following the identification of potentially modifiable risk and protective factors (Lemvigh et al., 2023).

Umbrella reviews are widely considered to be one of the highest levels of evidence synthesis currently available (Fusar-Poli and Radua, 2018). By reviewing previously published systematic reviews or meta-analyses, they allow for a uniform approach for all considered factors to permit their comparison. The best quality evidence for risk and protective factors for the development of a psychotic disorder are umbrella reviews that have classified the magnitude of effect for each factor, as well as the strength of the evidence for their association with psychosis risk (Arango et al., 2021, Radua et al., 2018), based on systematic reviews or meta-analyses. Umbrella reviews are not designed to test causality, only the association between factors and the outcome of interest. It is, therefore, outside their scope to explore the causal mechanisms of how identified factors modulate psychosis risk.

In this critical review, we will focus on causality by adopting the Bradford Hill criteria (Hill, 1965), leveraging strength, consistency and temporality from the umbrella reviews and integrating the remaining criteria (experiment, biological gradient, specificity, biological plausibility, coherence and analogy) to describe the putative mechanisms associated with these factors. This will allow us to gain understanding of how these risk and protective factors modulate psychosis risk and identify potential intervention targets, which is not possible from the results of umbrella reviews alone. We will first describe the underlying neurobiology of psychosis before appraising the distinct and shared putative causal mechanisms of the risk and protective factors identified with the strongest evidence for an association with psychosis onset.

## 2 Methods

As well as computing a magnitude of an effect, umbrella reviews categorise risk factors into classifications of strength of the association based on established criteria (Fusar-Poli and Radua, 2018): Class I (Highly convincing), Class II (Convincing), Class III (Suggestive), Class IV (Weak) and Non-significant. We included risk and protective factors meeting criteria for Class III strength of association with psychosis in published umbrella reviews of non-purely-genetic risk and protective factors for the onset of non-organic psychotic disorders (Arango et al., 2021, Radua et al., 2018). Class III risk and protective factors have evidence from over 1000 cases (plus further controls) and statistical significance of p<10^−3^, providing relatively strong evidence for a number of factors that provide a balance between breadth and focus for this review. In addition, any Class IV factors (significant associations) that were intrinsically linked to included factors were also included due to their overlapping mechanisms. For example, Black Caribbean ethnicity is considered a Class I factor, but other ethnicities are similarly associated with significantly increased risk for psychosis and have shared mechanisms but a lower strength of association (Class IV). This, therefore, led to the inclusion of a total of 25 risk factors (Table 1).

**Table 1:** Included factors and current evidence for causality from published umbrella reviews.

**Factor**	**Number of studies (cases)**	**Strength** **(eOR)**	**Consistency** **(CE)**	**Temporality** **(CES)**
Black Caribbean ethnicity in England	9 (3446)	4.87	I	IV
CHR-P status	9 (1226)	9.32	I	I
Obstetric complications	18 (1000)	1.97	I	NC
Cannabis use	10 (4036)	3.90	II	II
Childhood trauma	20 (2363)	2.87	II	IV
Ethnic minority in low ethnic density area	5 (1328)	3.71	II	IV
Impaired olfactory identification ability	55 (1703)	5.26	II	NC
Minor physical anomalies	13 (1212)	5.30	II	NC
Low premorbid intelligence	16 (4459)	2.12	II	IV
Second generation immigrant	26 (28,753)	1.68	II	IV
Stressful life events	13 (2218)	3.11	II	NC
Trait anhedonia	44 (1601)	4.41	II	NC
Childhood social withdrawal	15 (1810)	2.01	III	IV
Ethnic minority in high ethnic density area	5 (1328)	2.11	III	IV
First generation immigrant	42 (25,063)	2.19	III	IV
Non-right handedness	41 (2652)	1.58	III	ns
North African immigrant in Europe	12 (2577)	2.22	III	IV
Infection with Toxoplasma gondii	42 (8796)	1.82	III	IV
Urbanicity	8 (1328)	2.19	III	III
Winter/spring season of birth	27 (115,010)	1.04	III	NC
Asian ethnicity in England	6 (613)	2.83	IV	N/A
Black African ethnicity in England	4 (452)	4.72	IV	N/A
Mixed ethnicity in England	3 (330)	2.19	IV	N/A
Other White ethnicity in England	3 (274)	2.62	IV	N/A
Traffic/Pollution	1 (29)	5.55	IV	N/A

Abbreviations: CE, class of evidence; CES, class of evidence after sensitivity analysis only including prospective studies; eOR, equivalent odds ratio; N/A, not applicable; NC, not calculable (no prospective studies available to be analysed); ns, non-significant.

These individual risk factors were thematically organised according to overlapping concepts and mechanisms. Discussing each individual risk factor separately would have potentially skewed the content of the review and produced redundancies. Our thematic organisation allowed us to produce a cohesive critical review of the literature on mechanisms. Relevant articles were retrieved through international databases (PubMed, books, meetings, abstracts, electronic guidelines, and international conferences) and critically reviewed by the authors. Subsequently, results were presented after reaching a consensus (Table 2). This review is not following a systematic literature search, data extraction, or reporting approach, since our aim is to provide a conceptual perspective of the field.

**Table 2:** Summary of overarching mechanistic concepts, the environmental risk factors and signs that tie into them and the mechanistic pathways that lead to psychotic symptomatology.

**Overarching mechanistic concept**	**Environmental risk factors/signs**	**Mechanistic pathways**
Neurodevelopmental Abnormalities	• Impaired olfactory identification ability • Minor physical anomalies • Non-right handedness • Obstetric complications • Low premorbid intelligence	• Genes > Altered brain development > DA abnormalities & E/I imbalance • Infection > Inflammation > Altered brain development > DA abnormalities & E/I imbalance
Infection and the immune system	• Toxoplasma gondii • Winter/spring season of birth	• Infection > Inflammation > Altered brain development > DA abnormalities & E/I imbalance
Psychosocial Stress	• Childhood trauma • Ethnicity and ethnic density • Migrant status • Stressful life events • Urbanicity	• Psychosocial stress **>** HPA axis > Inflammation > Brain structure and function > DA abnormalities & E/I imbalance
Substance use	• Cannabis use	• Endocannabinoid system modulation > DA abnormalities & E/I imbalance
DA abnormalities	• Trait anhedonia	• DA abnormalities > Trait anhedonia
E/I imbalance	• Childhood social withdrawal	• E/I imbalance > Childhood social withdrawal
All above	• CHR-P state	• All above

Abbreviations: CHR-P, clinical high risk for psychosis; DA, dopaminergic; E/I, excitation/inhibition; HPA, hypothalamic-pituitary-adrenal

Firstly, we present neurochemical alterations hypothesised to underlie psychotic symptoms: i) altered dopaminergic signalling; and ii) excitation/inhibition balance.

Secondly, we explore mechanistic pathways that may contribute to these neurotransmitter alterations: i) neurodevelopmental abnormalities; ii) perinatal and chronic infection and the immune system; iii) psychosocial stress (incorporating ethnicity, migrant status and urbanicity); and iv) cannabis use.

Thirdly, we explore behavioural manifestations of neurobiological alterations: i) minor physical anomalies; ii) non-right handedness; iii) low premorbid intelligence; iv) impaired olfactory identification ability; v) trait anhedonia; and vi) childhood social withdrawal.

Finally, we discuss issues relating to the operationalisation of these risk factors, and the implications of our findings for clinical interventions designed to reduce the risk of later psychosis.

## 3 Neurochemical alterations in psychosis

### 3.1 Dopamine

Aberrant dopaminergic activity has the clearest evidence of a causal mechanism for psychosis. All licensed antipsychotic medications affect the central dopamine system, with a correlation between the affinity of antipsychotic medications for the D2 receptor and their clinical effectiveness (Richtand et al., 2007). The more effective an antipsychotic is at blocking the D2 receptor, the stronger its clinical effect. However, recent evidence suggests that the clinical efficacy of antipsychotic medication may also be mediated by modulation of D1 receptor activity (Yun et al., 2023).

Dopaminergic abnormalities are theorised to cause key psychotic symptomatology through aberrant salience and disruption of brain-wide signal integration (Howes et al., 2020, McCutcheon et al., 2019). Aberrant salience refers to the erroneous attribution of significance to internal or external stimuli that would normally be considered unimportant. This occurs due to the stimuli occurring closely in time to spontaneous dopamine signalling in the striatum (Kapur, 2003, Maia and Frank, 2017). Delusions are one of the core symptoms of psychosis and are characterised by unshifting conviction in unusual beliefs (Jaspers, 2013) and may be the result of aberrant salience. Once a stimulus is associated with spontaneous dopamine signalling, the stimulus may continue triggering dopaminergic activity, potentially reinforcing delusional beliefs (Saunders et al., 2018). In particular, dorsal regions of the striatum, the regions most strongly associated with abnormalities in psychosis, are associated with forming stable habits and beliefs (Everitt and Robbins, 2013, Kim and Hikosaka, 2013), as well as signalling threat-related information (Menegas et al., 2018). Aberrant dopaminergic activity in the striatum could, therefore, lead to the production of rigid, unshifting persecutory delusions (Howes et al., 2020). The striatum, particularly dorsal striatum, is also central in receiving and integrating signals from the whole cortex (Averbeck et al., 2014, Hunnicutt et al., 2016, McCutcheon et al., 2021) and moderating communication between limbic and motor regions (Lerner et al., 2015). Animal models have suggested that increased aberrant spontaneous phasic dopamine release, and a reduction in adaptive phasic release in response to relevant stimuli are important features of psychosis aetiopathology (Maia and Frank, 2017). This would lead to increased noise in dopamine signalling in the dorsal striatum, which could explain findings of reduced functional connectivity between the dorsal striatum and cortex (Fornito et al., 2013), and could disrupt integration of cortical inputs from emotional, cognitive, and motor areas.

The increase in striatal dopamine release is thought to underlie the dopamine-dependent positive symptoms of schizophrenia (Davis et al., 1991, Grace, 2016, Kapur, 2003, Krystal et al., 2017, Krystal and Anticevic, 2015, Lisman et al., 2008, Meltzer and Stahl, 1976, Modinos et al., 2015, Weinberger, 1987). However, this is unlikely to explain the negative and cognitive symptoms seen in psychosis.

### 3.2 Excitation/inhibition balance

Despite the strong neurobiological link between altered dopaminergic signalling and psychosis, conventional D2-blocking antipsychotics are not entirely effective in all patients, with around a third not adequately responding to treatment (Mailman and Murthy, 2010), even if D2 occupancy is high (Nordström et al., 1993). Therefore, even though dopaminergic alterations are key features of psychosis, the disorder is not explained by these alone (Moghaddam and Krystal, 2012, Stone et al., 2007). This is emphasised by the inadequate effects of antipsychotic medication on negative and cognitive symptoms (Mailman and Murthy, 2010).

Balanced excitatory (glutamatergic) and inhibitory (GABAergic) neural activity is required for synchronised neural oscillations (Uhlhaas and Singer, 2010, Yang et al., 2016). Disruptions in glutamatergic activity therefore may have brain-wide implications on function. In fact, negative and cognitive symptoms may be better explained by glutamatergic abnormalities but the evidence is inconsistent, particularly for negative symptoms (Javitt, 2010, McCutcheon et al., 2023, Merritt et al., 2021b, Moghaddam and Krystal, 2012). Antagonists of the glutamatergic *N*-methyl-d-aspartate receptor (NMDAR) (e.g. phencyclidine [PCP] and ketamine) induce positive, negative, and cognitive symptoms in healthy controls and exacerbate psychotic symptoms in individuals with schizophrenia (Krystal et al., 2003). Schizophrenia is associated with elevated levels of glutamatergic metabolites in the hippocampus, thalamus and striatum, and reduced levels in the anterior cingulate cortex (ACC) (Merritt et al., 2023b, Nakahara et al., 2022), which impact functional connectivity within the brain (Zahid et al., 2023b, 2024). Electroencephalography (EEG) studies suggest that elevated glutamate may compensate for early neurodevelopmental deficits (Krystal et al., 2017), reducing signal-to-noise discrimination of oscillatory brain activity and worsening hallucinatory symptoms (Adams et al., 2022). Greater variance in glutamate levels in psychosis patients compared to controls (Merritt et al., 2023b) may correspond with reports of higher ACC glutamate in patients with a poor therapeutic response to antipsychotics compared to treatment responders (Demjaha et al., 2014, Egerton et al., 2023, Egerton et al., 2021, Egerton et al., 2012, Fan et al., 2023, Iwata et al., 2019, Mouchlianitis et al., 2016), although this is not shown in all studies (Goldstein et al., 2015, Merritt et al., 2019, Tarumi et al., 2020, Zahid et al., 2022). These contrasting findings suggest that glutamate may play a complex and multifaceted role in the pathophysiology of the disease and its impact on treatment response (Merritt et al., 2021b).

Overall, a feedback loop between GABAergic and glutamatergic dysregulation and glutamate-induced excitotoxicity is thought to result in hippocampal hypermetabolism and atrophy (Lieberman et al., 2018, Manoach, 2003, Schobel et al., 2013). Post-mortem studies have shown that there are lower levels of glutamate decarboxylase 67 (GAD67), the enzyme that synthesises GABA, across cortical regions in psychosis patients (Curley et al., 2011, Guidotti et al., 2000, Hashimoto et al., 2008, Volk et al., 2000). Some studies show reductions of parvalbumin-positive GABA interneurons, particularly in the PFC and hippocampus (Beasley and Reynolds, 1997, Enwright et al., 2016, Hashimoto et al., 2003, Konradi et al., 2011), although the evidence is inconsistent (Tooney and Chahl, 2004, Woo et al., 1997). Lower numbers of parvalbumin-positive GABA interneurons in the ventral hippocampus lead to disinhibition of pyramidal cells, which in turn increases striatal glutamatergic activity (Gonzalez-Burgos and Lewis, 2012). The disinhibited pyramidal cells also disinhibit the nucleus accumbens (NAc), leading to increased inhibitory GABAergic innervation of the ventral pallidum.

These disruptions in GABAergic and glutamatergic signalling results in excitation/inhibition (E/I) imbalance that affects the efficiency of information processing across local and brain-wide circuits (Howes and Shatalina, 2022). The loss of parvalbumin positive interneurons, as observed in post-mortem examinations of schizophrenia (Kaar et al., 2019), results in a general disruption to the coordinated rhythmic oscillatory activity across the brain (Lewis et al., 2012, Lodge et al., 2009, Moreau and Kullmann, 2013) that orchestrates processing via cortico-cortical communications (Fries, 2009). Given that gamma oscillations are essential for cognitive processes and rely on effective parvalbumin positive interneuron function (Buzsáki and Wang, 2012), abnormalities observed in these frequency bands may explain the cognitive symptoms experienced in psychosis. Task related gamma oscillations generated by synchronised activity in the cortical pyramidal cells (orchestrated by parvalbumin-positive inhibitory interneurons (Cardin et al., 2009)) are shown to be consistently diminished in psychotic disorders, as measured by EEG studies (Reilly et al., 2018). However, results from investigations into spontaneous gamma activity at rest are less robust. While reduced resting-state gamma power correlates with psychotic symptoms and cognitive impairments in first episode psychosis (FEP) and schizophrenia, similar cognitive deficits and the presence of attenuated psychotic symptoms are associated with an increase in gamma and intact GABA concentrations in CHR-P, which may point to E/I-balance changes across stages of illness (Grent-’t-Jong et al., 2018). In addition, pre-clinical and human EEG/magnetoencephalography (MEG) studies investigating spontaneous resting-state gamma activity vary in their consistency and support for the E/I-balance model of schizophrenia; as schizophrenia does not replicate the same, established effects on gamma-band activity as ketamine, a psychotomimetic NMDA-R antagonist (Bianciardi and Uhlhaas, 2021).

Patients with psychosis also demonstrate sensory gating deficits (mediated by GABAergic receptors on glutamatergic neurons) (Daskalakis et al., 2007, De Wilde et al., 2007, Freedman et al., 2000) as well as impaired transcranial magnetic stimulation probed responses (mediated by GABAergic and NMDAR signalling) (Li et al., 2021) and mismatch negativity responses (NMDAR signalling) (Erickson et al., 2016). Computational models of EEG data have suggested that E/I imbalance may begin with a loss of synaptic gain on pyramidal cells, which then results in downregulated interneuron activity as a compensatory response (Adams et al., 2022).

This E/I imbalance also results in increased activity in midbrain dopamine neurons which project back to the dorsal striatum. Hyperactivity of the ventral subiculum may further disrupt i) function of the PFC, leading to cognitive deficits, and ii) function of the basolateral amygdala, leading to reduced emotional reactivity control, which may relate to negative symptoms (Grace, 2016, Grace and Gomes, 2019). As such, ventral hippocampal disruption could potentially contribute to the three main symptom dimensions of schizophrenia.

## 4 Mechanistic pathways

### 4.1 Neurodevelopment

A number of pre and perinatal complications have a small yet robust association with an increased risk of developing psychosis (Davies et al., 2020). Exposure to pre- and perinatal risk factors are associated with altered grey matter volume, with recent MRI studies highlighting enlarged striatal and reduced cingulate volumes (Holz et al., 2023b, Merritt et al., 2023a). As these regions (cingulate, insula, and striatum) contribute to salience processing, alterations in this network could increase an individual’s propensity to develop a psychotic disorder (Del Fabro et al., 2021, Palaniyappan and Liddle, 2012). Neurodevelopmental abnormalities can be associated with alterations in dopaminergic activity, particularly in the striatum (Davis et al., 1991, Grace, 2016, Kapur, 2003, Krystal et al., 2017, Krystal and Anticevic, 2015, Lisman et al., 2008, Meltzer and Stahl, 1976, Modinos et al., 2015, Weinberger, 1987).

Reinforcing this, perinatal insults in animal models, such as administration of methylazoxymethanol acetate (MAM) in pregnant rats, or lesioning the hippocampus in neonates, result in psychosis-like behaviour and pathology (Lodge and Grace, 2008, Oliver et al., 2020a). Neonatal lesioning of the hippocampus disrupts the development of cortical and subcortical networks that involve this region (Lipska and Weinberger, 2002, Mattei et al., 2015, Tseng et al., 2007) and alters functional connectivity between the hippocampus and prefrontal cortex (PFC) (Goto and O’Donnell, 2004, Lipska and Weinberger, 1998, O’Donnell et al., 2002). Reduced functional connectivity may impact synaptic pruning during early adolescence, which facilitates maturation of neural pathways by eliminating infrequently used synapses (Germann et al., 2021). PFC neurons may be subject to excessive pruning due to reduced signalling with the hippocampus, resulting in reduced length and spine density of PFC neurons, as observed in schizophrenia (Alquicer et al., 2008, Flores et al., 2005). During adolescence, synaptic pruning is particularly extensive in areas related to cognition (Cardozo et al., 2019, Huttenlocher and Dabholkar, 1997). This period of active synaptic pruning, and in particular excessive, aberrant pruning, is considered to be influential in psychosis aetiopathology (Feinberg, 1982), before synaptic density stabilises in adulthood (Shaw et al., 2008). These periods of active pruning may be started and ended by perineuronal nets (Fawcett et al., 2019), structures that surround neurons, particularly parvalbumin positive interneurons (Sorg et al., 2016). Research in CHR-P individuals suggests that there is a general disruption of processes like synaptic pruning, resulting in an altered trajectory of brain development in early adulthood (Chung et al., 2018, Merritt et al., 2021a). Synaptic pruning in individuals with psychosis is particularly disproportionate in the dorsolateral PFC (DLPFC) and hippocampus, which may lead to overactivation of mesostriatal dopaminergic pathways (De Bartolomeis et al., 2014). These changes in DLPFC may be partly explained by reduced DLPFC perineural nets in patients with schizophrenia (Alcaide et al., 2019).

### 4.2 Perinatal and chronic inflammation

While the brain was long considered to be an immune-privileged site (largely due to the blood-brain barrier), evidence of complex interactions between the brain and immune system has emerged. Immune cells are prevalent in meningeal lymphatic vessels and can play a role in regulating brain-wide networks (Filiano et al., 2016). Moreover, glia can directly affect neural structure and function, such as through synaptic pruning (Howes and McCutcheon, 2017). These findings lend support to the notion that the immuno-inflammatory system can contribute to neuroanatomical and neurofunctional changes. The most commonly-studied immune markers in psychosis research are peripheral blood cytokines, which are elevated in medication-naïve FEP patients (Pillinger et al., 2019), and markers of chronic low-grade inflammation (i.e., C-reactive protein) (Mondelli, 2014). Similarly, IL-6 and IL-4 are seen to be elevated in CHR-P compared to controls with baseline IL-10/IL-6 ratio and vascular endothelial growth factor (VEGF) also shown to be elevated in CHR-P individuals who transitioned to psychosis (Mondelli et al., 2023). Evidence for the effect of chronic low-grade inflammation on brain structure as measured by peripheral cytokines is mixed. Reduced grey matter volume has been associated with peripheral inflammation in regions such as the hippocampus, the orbital frontal cortex, the middle frontal gyrus, and the cingulate cortex (Kose et al., 2021). Other studies have found evidence for elevated brain measures associated with inflammation (Lizano et al., 2021, Lizano et al., 2019). Despite recent evidence for a genetically determined IL-6 association with brain structure (Williams et al., 2022), it seems that inflammation in psychosis has heterogeneous mechanisms at play that lead to pleiotropic expressions with distinct neuroanatomical signatures (e.g. Interferon-gamma [IFN-γ] associated with increased grey matter volume and IL-6 associated with reduced grey matter volume (Lalousis et al., 2023).

Infection of the mother during pregnancy can lead to changes in the foetal environment, which can influence the course of foetal brain development (Meyer et al., 2007), priming the brain and leading to vulnerability (Canetta et al., 2014; Cannon et al., 2014). As infections are more prevalent in colder months, this is more relevant for offspring born in the winter and spring in the northern hemisphere. Two prominent preclinical models of psychosis rely on infective probes during gestation in rodents: maternal gestational exposure to the human influenza virus, and administration of the viral mimic polyriboinosinic-polyribocytidilic acid (Poly[I:C]) (Oliver et al., 2020a). After prenatal immune activation, behavioural, cognitive and neurochemical changes arise in late adolescence or early adulthood (Ozawa et al., 2006, Piontkewitz et al., 2011, Vuillermot et al., 2010, Zuckerman et al., 2003), following the expected timeline of psychosis progression (Tandon and Fleischhacker, 2005). The severity of these alterations appears to be dependent on the intensity of a cytokine-mediated immune response (Meyer et al., 2005). There is no clear evidence that genetic liability to schizophrenia increases the likelihood of influenza infection or predisposes to a disrupted immune response to influenza (Leppert et al., 2019). However, winter/spring birth increases risk of perinatal infection due to seasonal changes in infection prevalence. Maternal-foetal transfer of pathogenic antibodies may be a mechanism in the development of psychosis (Wright and Murray, 1993). In preclinical models, transferring maternal antibodies to offspring leads to neuropathological and behavioural abnormalities (Brimberg et al., 2016, Coutinho et al., 2017, Jones et al., 2020). Furthermore, maternal-foetal transfer of recombinant NMDAR NR1 antibodies induced motor hyperactivity, and impaired sensorimotor gating, both of which are psychosis-like phenotypes (Jurek et al., 2019).

Both maternal influenza and poly(I:C) models display GABAergic abnormalities, particularly a decrease in reelin-positive GABAergic hippocampal neurons. Without reelin, lissencephaly (smooth brain) and cerebellar hypoplasia can occur (Hong et al., 2000). Reelin is crucial for neural positioning, synaptic plasticity as well as the acquisition, consolidation and expression of memory. These changes replicate the GABAergic abnormalities in the hippocampus that lead to downstream dopaminergic and glutamatergic dysfunction (Lieberman et al., 2018, Manoach, 2003, Schobel et al., 2013). The poly(I:C) model also displays structural abnormalities with poor axonal development and delayed myelination (Makinodan et al., 2008) also seen in patients with schizophrenia (Foong et al., 2002, Foong et al., 2000, Hakak et al., 2001, Lim et al., 1999, Sugai et al., 2004, Tkachev et al., 2003, Uranova et al., 2001). Behaviourally, these models also show behavioural abnormalities, including impaired prepulse inhibition, reduced exploration and reduced social interaction (Meyer et al., 2009, Shi et al., 2003), that mirror those in psychosis patients (Bolino et al., 1994, Braff et al., 1978, Braff et al., 1992, Catalan et al., 2021, Cornblatt et al., 2012, Perry and Braff, 1994).

In some patients, these inflammatory changes do not just present and resolve in early life and instead appear to be chronic, which is typically maladaptive and associated with tissue-specific or systemic pathology. Autoimmune encephalitis, particularly anti-NMDAR encephalitis, can present with acute psychosis in adults (Al-Diwani et al., 2019, Al-Diwani et al., 2017, Pollak et al., 2014) but still represents a relatively small proportion of psychosis cases (Kelleher et al., 2020), with increased serum NMDAR IgG not seen in the general psychosis population compared to controls (Cullen et al., 2021). The typical pattern includes prodromal malaise, or influenza-like symptoms, before the emergence of affective, cognitive and psychotic symptoms (Al-Diwani et al., 2019, Kayser et al., 2013, Titulaer et al., 2013). NMDAR antibodies are of particular interest in psychosis due to the glutamatergic abnormalities seen in the disorder. Anti-NMDAR encephalitis may be associated with influenza infection as its peak of incidence corresponds to peak influenza incidence in the winter (Pillai et al., 2015). Similarly, Māori and Pacific Island populations are more susceptible to severe influenza infection and have higher incidence and potentially more severe outcomes of anti-NMDAR encephalitis (Jones et al., 2017, Wilson et al., 2012). In some instances, patients who have tested positive for neuronal autoantibodies such as NMDARf antibodies, respond to immunotherapies and the case has been made that they form a separate diagnostic category of autoimmune psychosis (Pollak et al., 2020). In such cases the immunopathology is poorly understood. Generally it seems that there is minimal neuronal loss and low density of inflammatory cells (Bien et al., 2012). On the other hand, in cases of systemic low-grade inflammation and elevated proinflammatory cytokines, potentially resulting from genomic predisposition or response to environmental factors, may lead to activation of astrocytic dysfunction and/or microglia activation, resulting in dendritic pruning and synaptic changes (Corsi-Zuelli and Deakin, 2021).

#### 4.2.1 Toxoplasma gondii

Toxoplasma gondii is a parasite that can infect humans through contact with cat faeces or consuming undercooked meat (Wastling et al., 2000). If a mother is infected prenatally, Toxoplasma gondii can cross the placental wall and infect the foetus, affecting brain development through selective infection of muscle and brain tissue (Torrey and Yolken, 2003). Toxoplasma can increase the risk of psychosis through neurodevelopmental changes that only become apparent in late adolescence or early adulthood (Torrey and Yolken, 2003). However, Toxoplasma gondii infection can also result in psychotic symptoms acutely through encephalitis with affected individuals initially presenting with psychiatric symptoms with neurological symptoms only developing later (Kramer, 1966, Minto and Roberts, 1959).

### 4.3 Psychosocial stress

Both childhood trauma and experiencing stress in adulthood (for example, death of a loved one, serious illness or loss of employment) are robustly associated with psychosis (Radua et al., 2018). It has been estimated through the population attributable fraction (PAF) that 37.8% of schizophrenia cases could be prevented if childhood trauma were eradicated (Dragioti et al., 2022). Childhood trauma occurs at a time of substantial brain maturational changes (Merritt et al., 2021a), and so feasibly may contribute to a stress diathesis model of psychosis (Cullen et al., 2024).

The neural diathesis-stress model suggests that the hypothalamic-pituitary-adrenal (HPA) axis may be the primary physiological mechanism that mediates the relationship between psychosocial stress and psychosis (Pruessner et al., 2017, Pruessner et al., 2017; Walker et al., 2008; Walker and Diforio, 1997). When exposed to stress, the sympathetic branch of the autonomic nervous system is the first to respond by initiating adrenaline release, increasing heart rate, vasodilation and reducing digestion, ultimately engaging the HPA axis (Day and Pariante, 2012, Krugers et al., 2012). Subsequently, the HPA axis elicits corticotrophin-releasing hormone (CRH) release from the paraventricular nucleus of the hypothalamus and adrenocorticotropic hormone (ACTH) release from the pituitary gland. ACTH acts on the adrenal glands to induce glucocorticoid release (in humans, cortisol), which interact with different physiological systems in response to stress, having downstream effects on glucose metabolism, cardiovascular function, immune function and, most crucially, brain function (Walker et al., 2008). HPA axis activity is regulated by glucocorticoids binding to high-affinity mineralocorticoid receptors and low-affinity glucocorticoid receptors (Krugers et al., 2012) in the hypothalamus and pituitary, where they inhibit the secretion of CRH and ACTH by a process of negative feedback (Laryea et al., 2013).

The hippocampus, PFC, and amygdala have significant effects on glucocorticoid release and behavioural responses to stress, likely due to being rich in glucocorticoid receptors (Smith and Vale, 2006). Specifically, the hippocampus and PFC inhibit HPA axis activity and participate in the regulation of the HPA axis by means of glucocorticoid feedback inhibition. In contrast, the amygdala can engage the HPA axis when activated by emotional and physiological stressors. Abnormalities in these brain regions can therefore lead to dysregulation of the HPA axis and stress responses.

Brain structure and function have been shown to be correlated with stressors experienced during early life and these alterations overlap with psychosis aetiopathology. Adults with a history of childhood trauma display reduced grey matter in frontal and limbic networks (Calem et al., 2017; Frodl et al., 2017; Lim et al., 2014b; Paquola et al., 2016; Pollak et al., 2022; Tymofiyeva et al., 2022; Yang et al., 2023), a pattern which is also observed in psychosis patients (Kim et al., 2015, Picó-Pérez et al., 2022, Shepherd et al., 2012, van Erp et al., 2018). Additionally, there is some evidence of increased basal ganglia volume, which may be relevant to psychosis (Holz et al., 2023b, Merritt et al., 2023a, Popovic et al., 2020). These neurodevelopmental abnormalities can increase the sensitivity of the dopaminergic system, particularly in response to stress (Egerton et al., 2017, Egerton et al., 2016). Childhood trauma is also associated with significantly increased activation in the left superior frontal gyrus and left middle temporal gyrus, and decreased activation in the left superior parietal lobule and the left hippocampus (Heany et al., 2018). Resting-state functional connectivity (rs-FC) is also altered in people who have experienced early social adversity (Holz et al., 2023a). rs-FC between the amygdala and PFC, ACC and hippocampus are disrupted, with decreased rs-FC seen within both the frontoparietal and default mode networks (Holz et al., 2023a). The extent of alterations may be dependent on sex, with stress in early life being associated with reduced rs-FC between hippocampus-anterior cingulate in both adolescent females and males, but reduced amygdala-anterior cingulate rs-FC in females only (Herringa et al., 2013).

This could be, in part, explained by social defeat: the experience of belonging to the outgroup and feeling lesser (Selten and Cantor-Graae, 2005). Social defeat increases psychosis risk both directly (Björkqvist, 2001, Selten and Cantor-Graae, 2005) and potentially by mediating the relationship between stressful life events and psychosis onset (Selten et al., 2013). Social defeat has been shown to increase dopamine sensitivity in animal studies, likely through increased numbers of D2 receptors (Covington and Miczek, 2001, Morgan et al., 2002, Nader et al., 2006). Positron Emission Tomography (PET) studies in healthy controls have shown that dopamine release is proportional to salivary cortisol response following psychosocial or metabolic stress (Adler, 2000, Mizrahi et al., 2012, Pruessner et al., 2004). This elevated dopamine response is also seen in psychosis patients following stress exposure, suggesting a potential pathway from social stress and defeat to a sensitised dopaminergic system, eventually leading to psychosis onset.

The HPA axis has complex interactions with the immuno-inflammatory system and there is evidence to suggest that immune and inflammatory responses may mediate the effects of psychosocial stress on psychosis risk (Leboyer et al., 2016). For example, a stress-induced inflammatory response was observed in psychosis patients (characterised by selective release of cytokines, including IL-6), which was not seen in unaffected siblings or healthy controls (Corsi-Zuelli et al., 2020). However, childhood maltreatment was associated with increased TGF-β levels in both the patients and siblings but decreased TGF-β in controls (Corsi-Zuelli et al., 2020).

Studies conducted over the past five decades have shown that individuals with psychosis have higher concentrations of blood and salivary cortisol in comparison to healthy controls (Girshkin et al., 2014, Hubbard and Miller, 2019, Misiak et al., 2021). Conversely, cortisol responses to awakening and in response to psychosocial stressor tasks are attenuated among individuals with psychosis relative to controls (Berger et al., 2016, Ciufolini et al., 2014, Dauvermann and Donohoe, 2019, Zorn et al., 2017). It is hypothesised that this pattern may be indicative of chronic activation of the HPA axis (characterised by high basal/unstimulated cortisol levels), following repeated exposure to psychosocial stressors, which then reduces the ability of the HPA axis to mount an appropriate response when faced with acute stressors (Shah and Malla, 2015). In turn, an inadequate stress response may cause prolonged exposure to stressors and their negative sequelae and may engender less effective behavioural and psychological responses (Pruessner et al., 2013; Pruessner et al., 2017).

There is some evidence to suggest that chronic stress induces structural changes in brain regions regardless of life stage. Whilst low-stress or low-cortisol concentrations have particular neurotrophic effects, prolonged high stress (in dose or time) is thought to induce neurotoxicity by different mechanisms such as the glutamate cascade, the inhibition of glucose transport, and reducing brain-derived neurotrophic factor (BDNF) expression (McEwen et al., 2016). Childhood trauma and stressful life events appear to be associated with reduced BDNF mRNA levels in FEP patients (Mondelli et al., 2011). This may lead to decreased hippocampal volume through stress-induced IL-6 expression and cortisol release in three independent biological pathways (Mondelli et al., 2011).

Chronic exposure to stress hormones has been shown to adversely affect the brain structures involved in cognition and psychiatric disorders, where the timing and duration of the exposure is a crucial factor (Lupien et al., 2018, Lupien et al., 2009). Specific brain areas may be more sensitive to the effects of stress hormones during periods when they are undergoing development: For example, the hippocampus (which undergoes significant development during the first two years of life) may be more vulnerable to stressors occurring during very early life (Lupien et al., 2018) whereas stress exposure during late childhood and adolescence might lead to changes in amygdala volume and the frontal cortex, respectively (as these brain regions continue to develop up to these developmental periods). An early longitudinal study observed that diurnal cortisol levels were inversely correlated with left hippocampal volume in patients with FEP at both baseline and follow-up (Mondelli et al., 2010). Other studies have reported that a decreased cortisol awakening response is associated with reduced hippocampal volumes in males, both at the CHR-P ( Pruessner et al., 2017) and FEP stages (Pruessner et al., 2015). There is also some evidence that exposure to chronic high cortisol results in poorer cognition across various domains (Aas et al., 2019, Havelka et al., 2016, Labad et al., 2016), but this evidence is inconsistent (Aas et al., 2011, Cullen et al., 2014). Lower brain volume and poorer cognition could be due to early neurodevelopmental abnormalities influencing brain regions that are responsible for mediating HPA axis function and specific cognitive functions (i.e., hippocampus and prefrontal cortex) (Cullen et al., 2014). This may suggest differential effects of childhood trauma and the experience of stressful life events in later life.

#### 4.3.1 Ethnicity and migration

Evidence from Western studies has shown that individuals who are from ethnic minority backgrounds are at greater risk of developing psychosis compared to White individuals (Kirkbride et al., 2012, Radua et al., 2018, Tortelli et al., 2015). This risk is more pronounced when living in an area alongside relatively few other ethnic minority individuals (low ethnic density), but remains substantial even in high ethnic density areas (Bosqui et al., 2014, Radua et al., 2018). There has similarly been evidence of higher psychosis risk in first and second generation immigrants (Radua et al., 2018, Stroup et al., 2000), with even higher risk in refugees (Brandt et al., 2019). This is not exclusively ethnicity-based, with psychosis risk persisting in immigrants from White backgrounds (Kirkbride et al., 2012, Radua et al., 2018) and even White Europeans migrating within Europe or within their country (Morgan et al., 2019).

Ethnic minority and migrant status likely mediates its risk via psychosocial stress, partly linked to racism, xenophobia and discrimination (Anglin et al., 2021), as perceived discrimination is directly associated with psychosis (Anglin et al., 2014, Oh et al., 2014, Stowkowy et al., 2016). This discrimination can lead to social exclusion and isolation, exacerbated when an individual’s native language is more distant from the local language (Jongsma et al., 2021). The mechanisms that underly this elevated risk for psychosis likely overlap, with shared social factors feeding into neurobiological changes. Multiple theoretical models describe pathways through which racism induces psychosocial stress that accumulate over time and negatively affect health (Clark et al., 1999, Harrell, 2000). In many countries, structural racism has had an enduring effect on health by limiting an individual’s ability to access resources and control life circumstances, such as status and beneficial social connections (Phelan and Link, 2015).

Psychosocial stress may be a major explanatory mechanism for increased psychosis risk in these populations. Ethnic minority status intersects with exposure to a number of other risk factors described above, as ethnic minority populations are more likely to report exposure to various childhood adversities, such as childhood trauma (Grattan and Linscott, 2019), childhood maltreatment and exposure to violence (Roberts et al., 2011). Furthermore, patients with psychosis from ethnic minority backgrounds are more likely to report exposure to trauma and adversity compared to White, majority background patients, including physical abuse (Thompson et al., 2009), sexual abuse (Rosenberg et al., 2007), domestic violence (Rosenberg et al., 2007), separation from a parent (Poulton et al., 2014), and parental death (Rosenberg et al., 2007). This may lead to neurobiological changes, for example, people from ethnic minority backgrounds show increased amygdala reactivity to outgroup individuals (McCutcheon et al., 2018), which could be a potentially contributory mechanism given that they are exposed to outgroup individuals almost constantly.

Neural changes are pronounced in ethnic minority populations. Discrimination is correlated with higher amygdala activity and increased functional connectivity between the amygdala and other brain regions, particularly the thalamus (Clark et al., 2018), similar to findings in psychosis patients experiencing paranoid symptoms (Walther et al., 2022). Stress and trauma combined with low socioeconomic status throughout adolescence is linked to earlier completion of brain development and lower brain volume (Gur et al., 2019), indicating a neurodevelopmental link, either due to earlier curbing of synaptic density increases or greater synaptic pruning throughout adolescence. Migrants have been found to show elevated striatal stress-induced dopamine release and dopamine synthesis capacity compared to non-immigrants (Egerton et al., 2017). Moreover, discrimination is associated with paranoia in a dose-response fashion in clinical (Addington et al., 2007, Stowkowy et al., 2016) and non-clinical populations (Anglin et al., 2014, Combs et al., 2006, Kong, 2016). Informal and formal segregation and discrimination has led to neighbourhoods with higher proportions of individuals from ethnic backgrounds (Campbell et al., 1986, Massey, 1990) and inequitable access to clean air, healthcare, affordable childcare, education, safe housing and employment opportunities (Assari, 2018). These disadvantages are associated with cumulative stress (Walker et al., 2011), increasing psychosis risk. Residential instability, where people are living in multiple homes within one year, is predictive of earlier psychosis onset and neighbourhood disruption (Ku et al., 2020), where people fear being “pushed out” or that others have been “pushed out”, is associated with increased likelihood of psychotic experiences (Narita et al., 2020).

The higher incidence of psychosis in ethnic minorities may not be entirely explained by neurobiological changes. There is inequity in access to healthcare across ethnic groups and socioeconomic status (van der Ven and Kirkbride, 2018), meaning that care is delayed in adolescence and early adulthood, consequently increasing psychosis risk. It is important to note that ethnicity also includes social and psychological influences, including identity, explanatory models and belief systems about health and illness, levels of social support and social assets, and health risk behaviours which may also vary across ethnic groups (Zahid et al., 2023a). These may influence help-seeking, selection of preferred care providers, early recognition and intervention, and recovery, thereby increasing risk. Variations in these processes may explain ethnic inequalities of experiences and outcomes. There is additional evidence showing that some clinicians may tend to misinterpret symptom presentation in Black patients, overdiagnosing psychotic disorders and underdiagnosing mood disorders (Adebimpe, 1981, Gara et al., 2019, Mukherjee et al., 1983, Simon et al., 1973, Strakowski et al., 1997, Strakowski et al., 1996). Together, the relationship is not linear but rather a result of the interaction between multiple risk factors and conditions, with multiplicative or additive effects on outcomes.

The impact of inequitable healthcare provision on psychosis risk does not solely involve mental healthcare. Obstetric complications occur at higher rates in Black women compared to other groups (Giscombé and Lobel, 2005, Menon et al., 2011). These differences do not appear to be related to socioeconomic status or access to prenatal care (Lu and Halfon, 2003). Chronic stress induced by discrimination also potentially contributes to obstetric complications, that may go on to further heighten stress responses in offspring (Lipner et al., 2019). Stress during pregnancy increases the likelihood of preterm labour (i.e. prior to 37 weeks) and babies that are small for their respective gestational age (Copper et al., 1996, Davis et al., 2011, Dole, 2003, Hedegaard et al., 1996, Hoffman and Hatch, 1996). Black women with higher socioeconomic status have an increased risk of obstetric complications compared to White women from lower socioeconomic backgrounds (Lu and Halfon, 2003). While there is evidence that Latina women have lower birth complication rates following recent migration to the United States (Campos et al., 2008), the longer they stay in the United States, the higher the rates of obstetric complications, becoming progressively more similar to that of Black women (Fuentes-Afflick and Lurie, 1997, Premkumar et al., 2020). The evidence for immigrants more generally experiencing higher rates of obstetric complications is mixed (Ma and Bauman, 1996, Ortiz et al., 2019), but outcomes may be dependent on country of origin (Ma and Bauman, 1996, Von Katterfeld et al., 2012). Increased rates of obstetric complications may be caused by increasing cumulative experience of discrimination (Callister and Birkhead, 2002, Fox et al., 2015, Morales et al., 2002). Black women are more likely to have decreased cortisol during the second trimester of pregnancy compared to women from other backgrounds, which is consistent with women with posttraumatic stress disorder and lifetime stress exposure (Glynn et al., 2007). Cortisol is essential for foetal development, with lower cortisol in later stages of pregnancy associated with smaller foetuses in individuals who later develop schizophrenia (Ellman et al., 2019, Murphy et al., 2006, Trainer, 2002, Welberg et al., 2001). Black women are similarly shown to produce higher levels of other stress biomarkers (e.g. C-reactive protein, ACTH) during mid-to-late pregnancy compared to White women, controlling for socioeconomic status (Borders et al., 2015, Gillespie et al., 2016). Together, higher stress may be experienced by women from ethnic minority backgrounds, and this may lead to abnormalities in neurodevelopment and chronic exposure to stress hormones in offspring, thus increasing psychosis risk.

#### 4.3.2 Urbanicity and Traffic

Urbanicity, the impact of living in urban areas (Vlahov and Galea, 2002), is a risk factor for psychosis. This is an important risk factor to consider as currently 50% of the global population live in cities (United Nations Department of Economic and Social Affairs, 2019) and this is likely to increase further in the future.

Living in a city increases exposure to many other environmental and social risk factors for psychosis, indirectly increasing risk (Abrahamyan Empson et al., 2020). Exposure to infectious agents during pregnancy (Brown, 2011, Brown et al., 2004, Susser et al., 2000, Torrey et al., 2007, Yolken et al., 2009), obstetric complications (Eaton et al., 2000) and cannabis use (Coughlin et al., 2019) may be more likely to occur in urban environment. Immigrant and ethnic minority populations are also more likely to live in urban environments. As discussed above, psychosis risk is further elevated if an individual’s own ethnic group density is low (Boydell et al., 2001, Veling et al., 2008). Discrimination and “minority stress” may thus play a key role in increasing psychosis risk in urban populations (Gevonden et al., 2014).

However, psychosis prevalence in urban areas remains high after controlling for these variables, suggesting that specific characteristics of urban environments themselves may also directly influence psychosis risk (Harrison et al., 2003, Kuepper et al., 2011). This association has not been consistently shown in the Global South (DeVylder et al., 2018, Roberts et al., 2023), however, these data are based on cross-sectional point prevalence from self-report questionnaires rather than incidence from established ICD/DSM diagnoses so have limitations (Kirkbride et al., 2018).

An obvious difference between urban and rural areas is the availability of green spaces, which are generally associated with better mental health (Catalan et al., 2023, Coventry et al., 2021). There are differential patterns of neuronal activity in the PFC when exposed to urban or natural stimuli (Chen et al., 2016, Igarashi et al., 2015, Song et al., 2014). While the absence of noise, pollution and social stress may mediate its beneficial impact on psychological wellbeing, the restorative properties of the experience of nature may also be an important factor that deserves specific examination in schizophrenia (Tost et al., 2015). Higher levels of air pollution are another key difference between urban and rural settings that may increase psychosis risk. Air pollution affects the development of the brain in early life (Calderon-Garciduenas et al., 2014; Dadvand et al., 2015), so could impact on emerging neuropathology alongside neurodevelopmental processes.

Urban living may increase social stress. Aggressive encounters, bullying, exclusion or feelings of inferiority may be more likely in urban environments, which link into the concept of social defeat outlined above. Similarly, violent crime is more prevalent in urban environments than in rural environments. Traumatic and violent experiences, particularly in early life, may mediate the association between urbanicity and psychosis risk (Frissen et al., 2015, Newbury et al., 2016). Moreover, positive psychotic symptoms (Schreier et al., 2009), particularly paranoia (Bentall et al., 2012, Janssen et al., 2003), are often secondary to experiences of trauma.

The relationship between urbanicity and psychosis risk appears to depend on timing of exposure, with higher risk associated with being born and raised in urban environments than living there in later life (Marcelis et al., 1999, Pedersen and Mortensen, 2001). In fact, both individuals moving from more urban to more rural areas in childhood and those living in urban areas throughout their lives appear to be at increased risk of psychosis, which suggests a critical period of susceptibility whereby urban birth and early upbringing has an enduring impact on psychosis risk (Logeswaran et al., 2023). Moreover, individuals with high polygenic risk for schizophrenia appear to be more likely to live in urban environments (Colodro-Conde et al., 2018, Maxwell et al., 2021, Sariaslan et al., 2016), suggesting a form of intergenerational drift where people at increased risk move to more densely populated areas prior to disorder onset. This difference in risk may be indexed by differential structural and functional brain changes. These changes include increased HPA axis reactivity (Steinheuser et al., 2014), emphasising the role of increased social stress and social defeat. Early life urbanicity is associated with decreased grey matter volume in the DLPFC, a region associated with cognitive control (Haddad et al., 2015). Reduced DLPFC volume is seen in psychosis patients (Kim et al., 2015, Picó-Pérez et al., 2022, Shepherd et al., 2012, van Erp et al., 2018) and could impact function, potentially impairing recognition and automatic responses to cues in the environment and their associated contexts. Alongside the elevated subcortical dopamine release seen in psychosis (Abi-Dargham et al., 2000, Howes et al., 2012, Kumakura et al., 2007) this could lead to aberrant salience (Kapur, 2003) and delusion formation, defined as misinterpreting irrelevant stimuli (Corlett et al., 2007, Gray, 1998). This could be exacerbated by the high variety and intensity of auditory stimuli present in urban environments (Gottschalk, 1972, Landon et al., 2016, Wright et al., 2014). Patients with schizophrenia appear to have increased responses to noises from urban environments compared to healthy controls (Tregellas et al., 2009, Tregellas et al., 2007) and have more difficulty integrating auditory stimuli into a single coherent understanding, particularly when stimuli include negative or frightening emotional valence (El-Kaim et al., 2015). These stimuli in urban environments are less predictable and more intense than in rural environments and, over time, the cumulative impact of these stimuli appear to increase risk, potentially due to compensatory neural alterations (Light and Braff, 2003, McGhie and Chapman, 1961, Nelson et al., 2014, Postmes et al., 2014). Additionally, these noises can disrupt sleep (Jakovljević et al., 2006), which is a common issue in psychotic disorders (Waite et al., 2020). Together, these studies suggest a potential association between acoustic alterations and psychotic disorders, which could be exacerbated by city living.

### 4.4 Cannabis use

The association between cannabis use and psychosis has been recognised for centuries (Ayonrinde, 2020), with substantial and increasing evidence for a causal relationship (Vaucher et al., 2018). There is a dose-response relationship, with the greatest risks of psychosis observed in daily users (Marconi et al., 2016, Robinson et al., 2022) and those who use high-potency cannabis (i.e. higher THC concentrations) (Petrilli et al., 2022). Cannabis use during childhood and adolescence is particularly detrimental (Arseneault et al., 2002, Cass et al., 2014, Gruber et al., 2012, Kiburi et al., 2021). It has been estimated that eradicating cannabis use could prevent 9.7–12.2% of new psychosis cases worldwide (Di Forti et al., 2019, Dragioti et al., 2022), and several experts have recommended that it is a target for preventative interventions as it may be more amenable to intervention than most risk factors (Lemvigh et al., 2023).

∆9-tetrahydrocannabinol (THC) is responsible for the intoxicating effects of cannabis. THC is a partial agonist at the cannabinoid receptors (CB1 and CB2). The CB1 receptor is predominantly found in the central nervous system with the highest concentrations in the neocortex, basal ganglia, hippocampus, cerebellum, and anterior olfactory nucleus (Glass et al., 1997). CB1 receptors are predominantly pre-synaptic, occurring on the terminals of GABA and glutamatergic neurons and decrease neurotransmitter release when activated by endogenous ligands such as 2-arachidonoylglycerol and anandamide. The CB2 receptor was initially thought to be localized only in immune cells in the periphery (Piomelli, 2003), but has more recently also been found in the cerebellum, brain stem, astrocytes and microglia (Chen et al., 2017, Suárez et al., 2008), and may also modulate the activity of neural pathways relevant to psychosis (Cortez et al., 2020). Endocannabinoid transmission is finely tuned with precise mechanisms for local synthesis and degradation. At the synaptic level, endocannabinoid signalling has an important role in regulating synaptic plasticity (Fernandez-Espejo et al., 2009). Systematically, it has been shown to regulate important functions relevant to psychosis such as cognition, perception, sleep, mood, motivation and reward (Hillard, 2015, Lu and MacKie, 2016, Stasiulewicz et al., 2020).

Administration of THC can induce transient psychotic symptoms in healthy volunteers (D’Souza et al., 2004; Ganesh et al., 2020; Martin-Santos et al., 2012; Morrison et al., 2009) and exacerbate psychotic symptomatology in patients with schizophrenia (D’Souza et al., 2005, McGuire et al., 1994). As well as triggering positive psychotic symptoms such as paranoia, hallucinations and delusions, cannabis can increase negative symptoms (Morrison and Stone, 2011) and impair of hippocampal dependent cognitive functions, especially episodic and working memory (Curran et al., 2016).

Structural MRI studies have explored differences among cannabis users and non-users in brain structures implicated to psychosis, such as the hippocampus, amygdala, putamen, and the PFC, however evidence is mixed. A meta-analysis suggested that regular adult users have smaller hippocampus and orbitofrontal cortex than healthy controls, although these volumes were unrelated to duration of cannabis use or dose (Lorenzetti et al., 2019). A recent study using the IMAGEN dataset measured changes in cortical thickness of 704 individuals over ten years (Albaugh et al., 2023). In the group who started using cannabis in adolescence (14–19 years), the changes were most pronounced in dorsal and lateral portions of the PFC (Albaugh et al., 2023). In the group who started using in young adulthood, there were more differences in temporal, parietal and midline areas (Albaugh et al., 2023). Another study, using PET demonstrated that individuals with cannabis use disorder have significantly lower synaptic density in the hippocampus (D’Souza et al., 2021), similar to what is seen in psychosis (De Bartolomeis et al., 2014).

A large number of functional neuroimaging studies have provided further insight into the effects of cannabis during acute intoxication, withdrawal and after chronic use (Bloomfield et al., 2019). Acute intoxication with THC is associated with widespread alterations in regional brain activity, but the effect on psychotic symptoms is correlated with medial temporal and striatal activation (Bhattacharyya et al., 2009). Chronic use in adolescence results in brain-wide functional alterations, particularly relating to frontolimbic and frontostriatal connectivity (Lichenstein et al., 2022). As well as being implicated in psychosis, these regions continue to develop during adolescence and have a high concentration of cannabinoid (CB) receptors.

Some PET and single-photon emission computed tomography (SPECT) studies suggest that acute administration of THC leads to increased striatal dopamine release in healthy volunteers (Bossong et al., 2015, Bossong et al., 2009, Stokes et al., 2009), but others have not (Barkus et al., 2011) and all of the studies had small sample sizes. The opposite effects have been observed in dependent cannabis users, who instead have reduced striatal dopamine synthesis capacity and release (Bloomfield et al., 2014; Tomasi et al., 2015; Van De Giessen et al., 2017). Similarly, studies which have measured glutamate-derived metabolites have found that acute cannabis intoxication increases glutamate levels in the striatum (Colizzi et al., 2020), but that heavy cannabis users display reduced glutamate-derived metabolites in both cortical and subcortical brain areas, compared to healthy controls (Colizzi et al., 2016). Chronic cannabis use is associated with reduced striatal dopamine synthesis capacity, particularly in individuals with cannabis use disorder, (Bloomfield et al., 2014) but no abnormalities are seen in striatal dopamine release or in D2 receptor expression (Ghazzaoui and Abi-Dargham, 2014). This is the inverse of what is typically associated with psychosis risk, therefore the precise mechanism of chronic cannabis use increasing psychosis risk is unclear. To summarize, while the findings of acute cannabis administration studies are in keeping with those in people with psychosis (Howes and Kapur, 2009; Merritt et al., 2023b; Nakahara et al., 2022), individuals with cannabis dependence appear to have the opposite differences, so could represent a potential compensatory mechanism that does not occur in individuals who develop psychosis.

EEG has high temporal resolution and is therefore able to measure the synchronicity of neural oscillations across distributed brain regions (Skosnik et al., 2016). In schizophrenia, reduced synchrony of neural oscillations correlates with symptoms and cognitive dysfunctions (Uhlhaas and Singer, 2010). Synchronised communication between distributed cortical regions relies on temporal coupling of low (theta) and high (gamma) frequency oscillations. Specifically, gamma frequencies that modulate activity locally, are distantly modulated by theta oscillations, where gamma is nested into the phase of theta oscillations (Canolty et al., 2006). GABAergic interneurons are understood to coordinate these oscillations (Gonzalez-Burgos and Lewis, 2008) via endocannabinoid dependent mechanisms (Morrison and Murray, 2020). Exogenously administered cannabinoids bind to CB1 receptors indiscriminately, and can therefore disrupt synchronisation (Morrison and Murray, 2020; Skosnik et al., 2016) and cross-frequency phase coherence (Kucewicz et al., 2011). Furthermore, theta, alpha, and gamma power have been found to be decreased in heavy users compared to non-users in studies of evoked and resting state EEG, where the greater the cannabis use, the lower the EEG power (Edwards et al., 2009, Skosnik et al., 2006). Moreover, intravenous THC administration in healthy volunteers results in decreased theta power, bi-frontal theta coherence (a measure of synchronicity between electrodes) and gamma-band coherence, which were related to psychotic-like symptom severity (Cortes-Briones et al., 2015, Morrison et al., 2011). Gamma-band power and coherence is similarly reduced in psychosis (Reilly et al., 2018).

The role of other cannabinoids is poorly understood, though there has been extensive research into the effects of cannabidiol (CBD), the second most common psychoactive compound in cannabis. CBD is not intoxicating and may have therapeutic potential in psychosis (Chesney et al., 2021, Davies and Bhattacharyya, 2019) and cannabis use disorder (Freeman et al., 2020). CBD was thought to protect against the acute cognitive and psychotic-like effects of THC (Englund et al., 2013, Morgan et al., 2012), and the declining concentrations of CBD in high potency cannabis (Potter et al., 2018) was considered to be a factor for increased psychosis risk over time (Englund et al., 2017). However, more recent studies have shown that inhaled CBD does not acutely reduce THC-induced effects (Englund et al., 2022, Lawn et al., 2023). Therefore, THC concentration can be considered the major causal element of cannabis that increases psychosis risk.

## 5 Behavioural manifestations of neurochemical alterations

### 5.1 Minor physical anomalies

Minor physical anomalies are subtle abnormalities that indicate altered development of the mouth, eye, ear, head, hands and feet (Weinberg et al., 2007). These anomalies likely develop early in gestation (Jones et al., 2022, Warkany, 1971) and share their embryonic origin with the developmental processes of the brain (Jones et al., 2022). These minor physical anomalies have minimal aesthetic or functional impact. Despite this, they persist into adulthood and can be identified through simple visual examination.

Due to their embryonic origins, minor physical anomalies are potentially relevant to the neurodevelopmental hypothesis of schizophrenia. However, they could also be tied into a more holistic view of psychosis aetiopathology, with CHR-P individuals with high numbers of minor physical anomalies showing increased salivary cortisol, impaired visual memory and more disorganised symptoms (Mittal and Walker, 2007). This suggests that in addition to representing neurodevelopmental abnormalities, they may also be a marker for chronic stress and related aetiopathology.

### 5.2 Non-right handedness

There is a higher prevalence of non-right handedness in individuals with psychosis than in the general population (Hirnstein and Hugdahl, 2014). This could potentially relate to differences in brain lateralisation as handedness and brain asymmetry are strongly correlated (Rodriguez and Waldenström, 2008). Neuropsychological and neuroimaging findings suggest that there are abnormalities in lateralisation in schizophrenia (Crow, 2013). In particular, dopaminergic frontostriatal circuits implicated in psychosis are lateralised (Klimkeit and Bradshaw, 2006).

Various hypotheses have been put forward to explain brain asymmetry such as the left hemisphere lag (where the left hemisphere develops later than the right), the left hemisphere being especially vulnerable to insults, and differences in structural and functional specialisation between hemispheres (Geschwind and Galaburda, 1985). In humans, handedness seems to be established early in development, strongly influenced by genetics (Annett, 2013, McManus et al., 2013). Hand preference is displayed in utero, with foetuses preferentially moving their right arms and sucking their right thumbs more often than their left as early as 15 weeks, which is associated with later handedness (Hepper et al., 1991). Like in psychosis, there does not appear to be a single gene that determines handedness: it is a multigenic trait. None of the key genes involved with handedness development (Duboc et al., 2015) appear to overlap with those associated with schizophrenia in the polygenic risk score (Consortium, 2014), suggesting that any related mechanism that inflates psychosis risk occurs later in life.

There is some evidence that pathological left handedness, where individuals who were born right-handed shifted to left handedness due to early left-lateralised brain injury, may increase psychosis risk, particularly in epilepsy (Irwin and Fortune, 2014, Sherwin, 1981, Sherwin et al., 1982). These acute neurodevelopmental insults may contribute to psychosis aetiopathology alongside genetic risk.

Psychosis risk may be more associated with mixed-handedness, rather than left-handedness (Hirnstein and Hugdahl, 2014), with brain asymmetry also more strongly associated with mixed- than with left-handedness (Rodriguez and Waldenström, 2008). This brain asymmetry may also involve corpus callosum abnormalities (Rodriguez and Waldenström, 2008), which are commonly associated with psychosis (Whitford et al., 2010).

### 5.3 Low premorbid intelligence

Premorbid intelligence quotient (IQ) is an estimate of an individual’s level of intelligence before psychosis onset. FEP patients who have greater impairments in premorbid IQ do not display higher genetic risk for psychosis compared to other patients (Ferraro et al., 2023) although those with consistently low IQ have the greatest polygenic liability relative to controls. One possibility is that impaired premorbid IQ serves as a proxy indicator for compromised neurodevelopment (Koenen et al., 2009, Pogue-Guile, 1997). Low premorbid IQ is associated with psychosis risk, with numerous theories suggesting mechanisms through which normal or high premorbid IQ levels might be protective against psychosis. These mechanisms include enhanced resilience in the face of stressful life events (Koenen et al., 2009), with higher IQ being positively correlated with higher polygenic risk score for resilience and negatively correlated with polygenic risk score for schizophrenia in CHR-P individuals (He et al., 2021).

Similarly, the cognitive reserve hypothesis proposes that those with higher premorbid IQ are better able to cope with the impact of neurodevelopmental abnormalities either because of higher brain structural reserve or because of better functional capacity to use compensatory forms of neural processing. Better cognitive reserve is associated with fewer psychotic symptoms in patients with psychosis, either due to greater ability to shift conviction in symptoms or increased insight, leading to earlier help-seeking (Barnett et al., 2006). Higher cognitive reserve may also inhibit aberrant neural processing that mediates psychotic symptoms. This could potentially occur through neural degeneracy, the ability of brain structures or connections to adapt to perform identical or similar functions (Edelman and Gally, 2001). Greater degeneracy enables more efficient computations related to perception and action through heightened neural flexibility. Neural degeneracy is positively correlated with IQ with higher degeneracy increasing computational efficiency and neural flexibility.

Children with average or above-average intelligence naturally develop moderate levels of degeneracy across brain-wide neural circuits. These are not limited to those related to cognition. This may be protective against psychosis as any aberrant signalling in one circuit may result in compensatory signalling in others, neutralising the overall impact on function and attaining balance. Conversely, children with low IQ levels develop reduced levels of degeneracy, and are less able to compensate for neurodevelopmental abnormalities that may increase psychosis risk.

Another consideration is the effect of other interacting risk factors. For example, people who regularly use cannabis have impaired cognition and some studies suggest there is a respective decline in IQ (Curran et al., 2016). This means while there is a substantial neurodevelopmental component, IQ can be affected in adolescence and early adulthood by exogenous factors, some of which may independently increase psychosis risk.

### 5.4 Impaired olfactory identification ability

The primary olfactory cortex can be found in the medial temporal lobe, with the olfactory association cortex overlaps with the amygdala, hippocampus and orbitofrontal cortex (Cohen et al., 2012, Nguyen et al., 2010, Turetsky et al., 2009b), which are all structurally abnormal in psychosis (van Erp et al., 2018). The development of the olfactory system occurs within a period of increased vulnerability, when other midline structures are produced (Treloar et al., 2010), suggesting that impaired olfaction could be a marker of aberrant neurodevelopment. Patients with schizophrenia have been shown to have reduced olfactory bulb volume, which correlates with olfaction ability (Nguyen et al., 2011). Further to this, shallow sulcal depth in the olfactory bulb is characteristic of neural abnormalities during early gestation, and this is seen in patients with schizophrenia (Turetsky et al., 2009a). Olfactory sulcal depth is also correlated with orbital sulcal depth (Turetsky et al., 2009a), a brain region that develops later (Gimenez et al., 2006), potentially indicating that early insults may have longstanding effects, even in regions that should be largely unaffected.

Olfactory identification impairments may be associated with dopaminergic disruption. Olfactory function is an early sign of Parkinson’s disease (Dan et al., 2021), showing relatively strong diagnostic performance (Nielsen et al., 2018) and appears to deteriorate rapidly, possibly in line with disorder progression (Ercoli et al., 2022). Parkinson’s disease pathology is primarily related to hypo-dopaminergic function in the nigrostriatal pathway (Heng et al., 2023). Similarly, dopaminergic activity appears to have a key role in the development of the olfactory bulb and of olfactory neurons (Kamath et al., 2012). Areas strongly implicated in olfaction, such as the olfactory bulb, contain a high number of dopaminergic neurons (Kamath et al., 2012). Further to this, disruptions in dopaminergic activity, particularly in corticostriatal pathways, are implicated both in psychosis psychopathology but also olfactory identification impairments. This may reflect that impaired olfaction is a manifestation of underlying neurobiological changes in psychosis. As this impairment is seen in individuals meeting CHR-P criteria (Catalan et al., 2021), this may mirror the pattern of disorder progression seen in Parkinson’s disease (Dan et al., 2021) and be used to identify individuals earlier. However, it is important to note that, despite early reports of an association (Brewer et al., 2003), impairment is not significantly associated with transition to psychosis in CHR-P (Catalan et al., 2021).

### 5.5 Trait anhedonia

Trait anhedonia describes a consistently reduced ability to experience pleasure from typically pleasant stimuli (Chapman et al., 1976). Patients with schizophrenia have more severe trait anhedonia compared with healthy controls (Radua et al., 2018, Yan et al., 2012) which, particularly in social contexts, has been considered an important risk factor for psychosis (Lenzenweger, 2006).

The presence of anhedonia prior to psychosis onset may be indicative of attenuated dopamine dysfunction. Anhedonia emerges due to aberrant functioning of reward processing, which is largely mediated by dopaminergic signalling in the mesocorticolimbic pathway, which overlaps with key regions implicated in psychosis aetiopathology (Pizzagalli, 2022). This pathway starts from the ventral tegmental area (VTA), projecting to the ventral (NAc) and dorsal (caudate, putamen) striatum, and then runs to the orbitofrontal cortex (OFC), more dorsal aspects of the PFC, and various subregions of the anterior cingulate cortex (ACC) (Haber and Knutson, 2010, Knowland and Lim, 2018, Pizzagalli, 2014). Reward processing can be separated into discrete subcomponents, including reward responsiveness (anticipating and reacting to a reward), reward valuation (deciding whether a possible reward is sufficiently high value to exert necessary effort to attain it) and reward learning (encoding that a reward is better than expected and adapting reward valuation accordingly) (Der-Avakian and Markou, 2012, Husain and Roiser, 2018, Treadway and Zald, 2013).

Like in psychosis pathology, reward processing and its disruption in anhedonia are determined by alterations in dopaminergic signalling in the VTA, striatum and PFC. Structurally, anhedonia is associated with reduced tract number and volume in the left superolateral branch of the medial forebrain bundle (Bracht et al., 2022, Bracht et al., 2014), which connects the VTA to the PFC. Increased structural connectivity between the VTA and medial PFC is associated with more severe anhedonia (Bracht et al., 2022), suggesting that this may be a mechanism to compensate for reward processing abnormalities. Similarly, reduced striatal (in particular, dorsal) and OFC volume are associated with anhedonia (Auerbach et al., 2017, Pizzagalli et al., 2009) and increased genetic risk of anhedonia (Howard et al., 2019).

Functionally, animal models of anhedonia are associated with reduced dopaminergic transmission in the ventral striatum, contrasting with increased dopaminergic transmission in the VTA and medial PFC (Pizzagalli, 2014). In particular, rodent models of depression present with anhedonia and reduced goal-directed behaviours to increased phasic bursting and excitability of dopaminergic VTA neurons (Cao et al., 2010, Han and Nestler, 2017, Knowland and Lim, 2018, Lowes et al., 2021). Similarly, optogenetic inhibition of dopaminergic neurons connecting VTA and NAc has been shown to reverse anhedonia elicited by chronic social defeat (experiencing aggressive encounters) (Chaudhury et al., 2013). These systems and directions of signalling overlap with the dopaminergic abnormalities seen in psychosis. There is some evidence that some antipsychotic medications may reduce the severity of psychotic symptoms through blocking GABA_A_ receptors on GABAergic neurons in the VTA (Lu et al., 2023), inhibiting dopaminergic innervation of the NAc, which in turn leads to disinhibition of the ventral pallidum and decreased dopaminergic activity in the midbrain which projects back to the dorsal striatum, overall reducing pathology.

This is echoed by studies in humans, with reduced dorsal (e.g., caudate, putamen) and NAc activation and reduced perigenual ACC activation seen in depressed patients during tasks assessing reward consumption (Forbes et al., 2006, Pizzagalli et al., 2009), reward anticipation (Borsini et al., 2020, Pizzagalli, 2014, Pizzagalli et al., 2009, Zhang et al., 2013), and reward learning (Gradin et al., 2011, Kumar et al., 2018, Kumar et al., 2008, Rupprechter et al., 2020). Reduced ventral striatal activation to receiving a reward is associated with anhedonia (Borsini et al., 2020), whereas larger reward prediction error signals (indexing greater reward learning) in the ventral striatum predicted reduced anhedonia after six months of follow-up (Eckstrand et al., 2019). Reduced activation in areas of the OFC related with reward is associated with more anhedonic symptoms in adolescents with depression (Xie et al., 2021). Lower functional connectivity between the caudal vmPFC and various reward regions (NAc, VTA, OFC) while listening to pleasant music is associated with greater anhedonia (Young et al., 2016). Reduced ventral striatal activity while anticipating reward is associated with anhedonia (Morgan et al., 2013, Stringaris et al., 2015) and may result in compensatory medial and dorsolateral PFC overrecruitment during reward processing (Forbes et al., 2009, O’Callaghan and Stringaris, 2019, Pan et al., 2017). Frontostriatal abnormalities have also been shown at rest, with higher left ventral striatal intrinsic FC at baseline increasing anhedonia (but not low mood) at age 14 (Pan et al., 2022). Aberrant high FC between the ventral striatum and the rest of the reward network may reflect an inability to modulate responses to reward-related cues in the environment. These resting state changes may be the result of interaction with inflammatory mechanisms. In a dopaminergic pharmacological challenge study with L-DOPA, reduced anhedonia following L-DOPA administration correlated with rsFC post-administration but only in patients with CRP > 2 mg/L (Bekhbat et al., 2022).

### 5.6 Childhood social withdrawal

Social functioning deficits during childhood are robustly associated with development of a psychotic disorder (Matheson et al., 2013, Radua et al., 2018). The core mechanisms for social withdrawal may stem from excitation/inhibition imbalance. Deficits in glutamatergic and GABAergic transmission is associated with negative symptoms (Merritt et al., 2013) and social deficits in psychosis (Han et al., 2014, Yizhar et al., 2011). It is thought that social withdrawal can exacerbate symptomatic development in psychosis, for example attributing false social meaning to hallucinations or delusions with social valence (Hoffman, 2007), potentially through aberrant salience.

These social cognition deficits impact social support. Patients with schizophrenia have less extensive social support networks, and this is seen from the early stages of the disorder, with smaller social networks seen in the CHR-P (Robustelli et al., 2017, Ryan et al., 2023) and FEP (Gayer-Anderson and Morgan, 2013) stages. In CHR-P it appears that social network size is more related to social cognition and social anxiety rather than paranoia or social anhedonia (Ryan et al., 2023) and social deficits do not improve if attenuated positive psychotic symptoms improve (Cornblatt et al., 2003, Ryan et al., 2023), which suggests that social withdrawal may be independent of symptomatic onset.

Greater social withdrawal is observed in children with psychotic-like experiences compared to those at increased genetic risk for psychosis onset. This suggests that there is a greater impact of early life experiences on social withdrawal. It is also possible that social withdrawal may contribute to, or be exacerbated by, the development of other early symptoms (Bradley, 2000; Hoffman, 2007).

## 6 The clinical high risk for psychosis state

The criteria for the CHR-P state include attenuated positive psychotic symptoms and a recent drop in functioning (Fusar-Poli, 2017, Yung et al., 2005). In addition to this, there is an accumulation of risk factors for psychosis compared to the general population (Fusar-Poli et al., 2017). From the mechanistic pathways discussed in this review, this accumulation of risk factors for psychosis is suggestive of progressive neurobiological changes over time, resulting in presenting attenuated psychotic symptoms. CHR-P individuals have altered brain structure (Luna et al., 2022, Merritt et al., 2021a) and function (Luna et al., 2022) compared to healthy controls. There is some evidence that these alterations in CHR-P represent an intermediate stage between healthy controls and FEP (Kim et al., 2022, Kindler et al., 2018, Korda et al., 2022), but this evidence is inconsistent (Oliver et al., 2023, Schifani et al., 2017, Zhao et al., 2022).

**Fig. 1:**
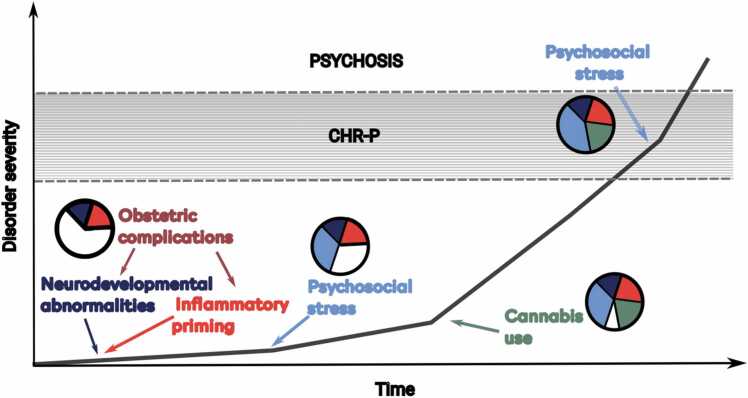
Summary figure illustrating a hypothetical potential trajectory to psychosis onset. This represents a single potential causal pie of many and may not represent an accurate portrayal of disorder progression, with included risk factors, pathways and their effects being purely illustrative. In this example, the mother of this individual experienced obstetric complications during pregnancy leading to neurodevelopmental abnormalities and inflammatory priming, increasing psychosis risk but not enough to lead to psychosis onset. Psychosocial stress led to another increase in psychosis risk before cannabis use led to further increased risk. The resultant changes led to the onset of attenuated psychotic symptoms, drop in global functioning and CHR-P onset. Further psychosocial stress increases risk and resulted in transition to psychosis. This hypothetical causal pathway is one of many potential trajectories to psychosis onset.

This follows a causal pie model, wherein an outcome may be caused by multiple causal pies composed of different component causes. A component cause (e.g. psychosocial stress) leads to brain changes but not severely enough to induce symptomatology. Only when a sufficient number of component causes are present, is a causal pie complete leading to the requisite brain changes to result in psychosis onset (Wensink et al., 2014) (Fig. 1). For example, perinatal infection could lead to hippocampal abnormalities that are exacerbated by living in an urban environment, as well as childhood trauma and heavy cannabis use that eventually trigger psychosis onset. Without one of these component causes, psychosis onset will not occur. However, it is currently unclear which component causes need to co-occur to induce psychosis onset.

Further complicating this picture, the same risk factors for psychosis onset in the general population may not be the same in CHR-P. For example, while cannabis use has robust association with psychosis risk in the general population, it does not appear to increase psychosis risk in CHR-P (Chester et al., 2023, Farris et al., 2020). This could be related to the timing of exposure, with cannabis use in adolescence being more impactful. Alternatively, it may be that different factors are more relevant for individuals at this level of increased risk compared to the general population. Currently the factors with the highest strength of evidence for increasing psychosis risk in CHR-P do differ from those in the general population, instead relating to symptomatic and functional impairment at first presentation (Andreou et al., 2023, Oliver et al., 2019b). This may mean that there are separate causal pies for CHR-P and psychosis. Considering the heterogeneity in the CHR-P population, there may alternatively be a large range of causal pies with varied component causes already present at this stage. This would provide low statistical power to determining exposures that cause psychosis onset.

## 7 Considerations for future research

### 7.1 Consider interactions between factors

Historically, risk and protective factors have been considered independently when, in reality, they interact with one another (Pries et al., 2021), potentially acting synergistically exacerbating common pathways of aetiopathology. In this review, we have provided evidence that several risk factors for psychosis operate on the same mechanistic pathway. For example, the effects of ethnicity, migrant status and urbanicity are likely all mediated through increased psychosocial stress. This complexity of interacting factors has been explored in the syndemics theoretical framework, which models the clustering of social and health problems at a population level (Zahid et al., 2023a, 2024). This could be explored quantitatively in future studies through complex pattern recognition and network analyses. The same risk factor may have different effects depending on the circumstances, particularly with some risk factors being shared across mental disorders (Arango et al., 2021), interactions between factors could help disentangle disorder onset across mental disorders (Oliver, 2024). Understanding these multifaceted relationships is not only crucial for effective prevention and intervention strategies but also highlights the need for a more comprehensive approach to healthcare that goes beyond mental health alone.

### 7.2 Explore universal and subgroup-specific risk and protective factors

Our understanding of psychosis has evolved over time, with distinct phases of investigation. Early research focused purely on schizophrenia with a subsequent shift to psychotic disorders more generally. This has impacted on the types of risk and protective factors identified. We have attempted to mitigate against this by selecting factors that modulate risk of psychotic disorders, not just schizophreniform psychoses. As well as broadly exploring psychosis risk, it may also be that there are distinct subgroups with specific associated factors. This could relate to differences in diagnoses (e.g. schizophreniform psychoses, mood disorders with psychotic features, schizoaffective disorder) or sub-populations within and across diagnoses. A better understanding of different presentations and their respective underlying component causes may help improve our ability to predict their onset.

### 7.3 Improve understanding of temporality and risk dynamics

Umbrella reviews are able to re-evaluate the evidence for risk factors through sensitivity analyses restricted to prospective studies. This allows us to consider the temporality of risk factors as prospective studies by establishing that exposure predates psychosis onset, without concerns about recall bias. However, the timing of these exposures may be more complex. Differential timing of exposures can lead to different neurobiological changes. For example, neighbourhood disadvantage in early childhood increases amygdala reactivity to neutral faces, an effect not seen with exposure in adolescence (Gard et al., 2021, Lawson et al., 2017). Charting the key risk periods for each risk factor and the specific scenarios that increase vulnerability to them is important for improving understanding of psychosis risk. As well as improving the ability to detect individuals at risk and predict outcomes, it could inform the design of clinical interventions to support people in key phases of vulnerability.

Similarly, the temporality of psychosis onset differs, with peak age of onset of psychotic disorders being 20.5 years of age (Solmi et al., 2022) but the risk can increase later in life, for example in individuals with anti-NMDAR encephalitis (Al-Diwani et al., 2019) or women during the perimenopausal period (Culbert et al., 2022). As the timing of the onset of these disorders differs, the risk factors associated with their onset likely differ too. More research is needed to understand which risk factors may be more relevant for these disorders and when they are most impactful.

### 7.4 Standardise operationalisation of risk and protective factor exposure

One of the limitations of umbrella reviews is that there is high heterogeneity of how risk and protective factors are measured and operationalised. For example, childhood trauma assessed prospectively during childhood and retrospectively in young adulthood yield exposed subgroups that typically do not overlap (Newbury et al., 2018). This may be due to children not recognising trauma at the time, and children or adults not disclosing past experiences when interviewed in later life. This issue could be addressed by studies collecting both types of data.

Similarly, there are multiple measures for assessing current cannabis use, including frequency of use, grams of cannabis per day, potency of cannabis used, and type of cannabis product used. The lack of standardisation across studies, and the unreliability of self-report compromise the assessments. Cannabis use appears to have a dose-response relationship with psychosis risk, so measures of lifetime cannabis exposure are worthwhile (Robinson et al., 2022). However, the accuracy of self-reported use is often unreliable, particularly in populations with psychosis (Chesney et al., 2023) and it has been proposed that using quantitative biological measures, such as urine or plasma carboxy-THC may, provide a more accurate guide of current use.

Furthermore, increasing standardised, regular measurements of potential risk and protective factors could be extremely informative. Some risk factors may be causally implicated but not sufficiently measured in research to be meta-analysed and therefore included in an umbrella review. For example, stimulants (e.g. cocaine, methamphetamine) directly increase extracellular dopamine (Kahlig and Galli, 2003) and can induce psychosis (Arunogiri et al., 2018, Sabe et al., 2021), yet does not have available meta-analytic evidence for the association between the exposure and psychosis. Moreover, the current evidence base for protective factors is relatively sparse with no significant protective factors identified in umbrella reviews (Arango et al., 2021, Radua et al., 2018). Protective factors are important for the ascertainment of good outcomes for patients (Petros et al., 2020) and modifiable protective factors could be important targets for future preventive interventions (Han et al., n.d, Lemvigh et al., 2023).

Overall, future longitudinal studies should try to harmonise their measures with previous and ongoing studies to encourage comparability and combining of datasets. This would result in datasets with greater statistical power that may be able to address some of the outstanding key questions outlined above to advance preventive psychiatry.

### 7.5 Development of preventive interventions targeting risk and protective factors

Understanding the complexities of risk and protective factors can inform the design of interventions designed to reduce the risk of psychosis onset. There are currently no published trials testing the effectiveness of universal preventive interventions, aimed at the entire population, to reduce the incidence of psychotic disorders (Brodeur et al., 2024). While testing universal preventive interventions for reducing psychosis incidence is logistically challenging due to the relatively low incidence of psychotic disorders in the general population, interventions reducing incidence or exposure to known causal risk factors may be an informative alternative. Targeting modifiable risk and protective factors, such as childhood trauma or cannabis use, could be used to reduce the population-level incidence of psychosis (Han et al., n.d.; Lemvigh et al., 2023). These interventions could also be adapted to individuals at increased risk due to other environmental exposures (i.e. selective interventions) or due to meeting CHR-P criteria (i.e. indicated interventions). Indicated interventions allow for increased intensity of intervention compared to universal interventions due to the increased risk, allowing for a lower number-needed-to-treat.

## 8 Conclusion

Research into psychosis risk has primarily focused on single risk and protective factors in isolation. This review indicates that many of the main risk factors for psychosis are mediated by common causal mechanisms. It is still unclear which particular combination of these factors is critical to cause psychosis onset. Future research focusing on understanding the shared covariance between these factors is essential for improving the detection of individuals at risk, prognostication of their outcomes and development of effective preventive treatments.

## Funding/Support

This study is supported by a Wellcome Trust grant (215793/Z/19/Z) to PFP. RAM’s work is funded by a Wellcome Trust Clinical Research Career Development (224625/Z/21/Z).

## Conflict of Interest Disclosures

AEC has received consultancy fees from Stratenym Inc and Symmetron Ltd. PAL has received honoraria for talks presented at educational meetings organised by Boehringer-Ingelheim outside of the current study. RAM has received speaker/consultancy fees from Karuna, Janssen, Boehringer Ingelheim, and Otsuka, and co-directs a company that designs digital resources to support treatment of mental illness. PFP has received research fees from Lundbeck and received honoraria from Lundbeck, Angelini, Menarini and Boehringer Ingelheim outside of the current study.

## Role of the Funder/Sponsor

The funder had no role in the design and conduct of the study; collection, management, analysis, and interpretation of the data; preparation, review, or approval of the manuscript; and decision to submit the manuscript for publication.
